# The Japanese version of the questionnaire about the process of recovery: development and validity and reliability testing

**DOI:** 10.1186/s12888-017-1520-y

**Published:** 2017-11-07

**Authors:** Akiko Kanehara, Risa Kotake, Yuki Miyamoto, Yousuke Kumakura, Kentaro Morita, Tomoko Ishiura, Kimiko Shimizu, Yumiko Fujieda, Shuntaro Ando, Shinsuke Kondo, Kiyoto Kasai

**Affiliations:** 1 Department of Neuropsychiatry, Graduate School of Medicine, The University of Tokyo, 7-3-1 Hongo, Bunkyo-ku, Tokyo, 113-8655 Japan; 2 Seiwa-Kai Nishikawa Hospital, 293-2 Minatomachi, Hamada-shi, Shimane, 697-0052 Japan; 3 Department of Mental Health /Psychiatric Nursing, Graduate School of Medicine, The University of Tokyo, 7-3-1 Hongo, Bunkyo-ku, Tokyo, 113-0033 Japan; 4 Department of Rehabilitation, The University of Tokyo Hospital, 7-3-1 Hongo, Bunkyo-ku, Tokyo, 113-8655 Japan

**Keywords:** Factor analysis, Questionnaires, Recovery, Reliability, Validity

## Abstract

**Background:**

Personal recovery is increasingly recognised as an important outcome measure in mental health services. This study aimed to develop a Japanese version of the Questionnaire about the Process of Recovery (QPR-J) and test its validity and reliability.

**Methods:**

The study comprised two stages that employed the cross-sectional and prospective cohort designs, respectively. We translated the questionnaire using a standard translation/back-translation method. Convergent validity was examined by calculating Pearson’s correlation coefficients with scores on the Recovery Assessment Scale (RAS) and the Short-Form-8 Health Survey (SF-8). An exploratory factor analysis (EFA) was conducted to examine factorial validity. We used intraclass correlation and Cronbach’s alpha to examine the test-retest and internal consistency reliability of the QPR-J’s 22-item full scale, 17-item intrapersonal and 5-item interpersonal subscales. We conducted an EFA along with a confirmatory factor analysis (CFA).

**Results:**

Data were obtained from 197 users of mental health services (mean age: 42.0 years; 61.9% female; 49.2% diagnosed with schizophrenia). The QPR-J showed adequate convergent validity, exhibiting significant, positive correlations with the RAS and SF-8 scores. The QPR-J’s full version, subscales, showed excellent test-retest and internal consistency reliability, with the exception of acceptable but relatively low internal consistency reliability for the interpersonal subscale. Based on the results of the CFA and EFA, we adopted the factor structure extracted from the original 2-factor model based on the present CFA.

**Conclusion:**

The QPR-J is an adequately valid and reliable measure of the process of recovery among Japanese users with mental health services.

**Electronic supplementary material:**

The online version of this article (10.1186/s12888-017-1520-y) contains supplementary material, which is available to authorized users.

## Background

The concept of person-centred care is essential for mental health services [1]. Subjective outcome measures are increasingly popular alongside measures of symptomatic remission or objective functioning [2]. Western mental health policies and studies have focused on personal recovery as an important clinical outcome [3, 4]. Personal recovery has been described as a unique process of changing one’s attitudes, values, feelings, goals, skills, and/or role and developing new meaning and purpose in life beyond the limits imposed by one’s illness [5]. The concept of recovery extends beyond removal of all symptoms or the complete restoration of functionality. Recovery involves minimising the impact of mental illness and maximising well-being (e.g., by developing valued social roles) [6]. A recent systematic review supported a conceptual framework of personal recovery consisting of the following factors: Connectedness; Hope and optimism about the future; Identity; Meaning in life; and Empowerment (abbreviated to CHIME) [7].

Effective recovery assessment tools are needed for better recovery-oriented support. A recent review found that the Recovery Assessment Scale (RAS) has been the most widely used [8, 9]; however, the Questionnaire About the Process of Recovery (QPR) is the only measure whose items all map onto the CHIME recovery framework [10, 11].

Service users, researchers, and clinicians collaboratively designed the QPR; it has high internal consistency reliability and validity [12]. An exploratory factor analysis (EFA) of the original 22-item version identified two factors; these were labelled ‘intrapersonal’ and ‘interpersonal’. Subscales examining these factors showed good internal consistency (intrapersonal: α = 0.94; interpersonal: α = 0.77) and good construct validity and reliability [10]. A 15-item version has also been developed [12] that is less burdensome and slightly more robust [13]. Community-level recovery-promotion intervention research (REFOCUS) and pre-post-evaluation of Recovery College students have used the QPR as a primary outcome measure [14, 15]. The 22-item Chinese version showed good validity and reliability as a measure of perceived levels of recovery [16].

Mental health service reform in Japan has been slower than in the other OECD countries, with psychiatric hospital care being dominant [17–19]. Japan requires community-based care and recovery-oriented services. Some person-centred service models were originally developed in Japan [20, 21]; however, no research has examined personal recovery using a validated assessment tool for users participating in a specific mental health service in Japan.

Accordingly, this study aimed to develop a Japanese version of the QPR (QPR-J) and examine its validity and test–retest and internal consistency reliability. We also tested the possible factor structures of personal recovery among Japanese service users using EFA with the QPR-J.

## Methods

### Study designs

The study was performed in two stages, using a cross-sectional and prospective cohort study design. Specifically, the first stage used a cross sectional design to develop and validate the QPR-J in users of mental health services, while the second stage used a prospective cohort design to evaluate the test-retest reliability of the tool in a subset of the participants.

#### Participants

We conducted a survey of mental health-related service users using the Japanese version of the QPR (QPR-J). The sample was recruited from 14 community mental health services in the suburbs of Tokyo. All the participants recruited from community mental health services had been diagnosed with mental illness and/or mental disorders by a psychiatrist. In our survey, the participants selected their own diagnosis, including the categories ‘other’ and ‘not known’, by themselves (self-report). Inclusion criteria were as follows: (1) aged ≥18 years, (2) able to participate and give informed consent, and (3) having any mental disorder. We explained the study to the participants. All participants provided a written indication of informed consent. Participation was voluntary. This study was approved by the Ethical Committee of the Faculty of Medicine, The University of Tokyo [approval No. 10890].

#### Development of the Japanese version of the QPR

The original QPR contains 22 items. Responses were made on a 5-point Likert scale (0 = *strongly disagree*, 4 = *strongly agree*). Scores’ possible range is 0–88. The intrapersonal and interpersonal subscales contain 17 and 5 items, respectively (possible score ranges: 0–68 and 0–20). Higher scores indicate greater recovery.

The following procedure was used to translate the QPR [22]. First, we obtained permission to translate the QPR from its original authors. Three native Japanese speakers independently translated the original instrument into Japanese. Eleven mental health professionals reviewed the three translations and collaborated to create a draft translation of the QPR-J. Second, the draft version was back-translated into English by a professional translator who was a native English speaker following the guidelines for the translation and adaptation process for patient-reported outcomes measures [22]. Then, another native professional translator checked the back-translation of the QPR. We refined the draft after we compared the original questionnaire with the back-translation. Third, the authors of the original QPR and the authors of the present study revised the back-translation based on the original concept. Fourth, we conducted pre-tests for users and peer staffs to confirm whether the items on the pre-final questionnaire were subjectively relevant and appropriate to the participants. We asked them what they thought about the items after answering the questionnaire. The participants reported that the items were easy to understand and relevant to their life situation. The survey used the finalised QPR-J; surveying ran from October to December 2015 (Additional file 1).

#### Validity testing

The Recovery Assessment Scale (RAS) is closely correlated with the original QPR [13]. Quality of life (QOL) is closely correlated with recovery [23]. Accordingly, the RAS and a scale measuring QOL were used to examine the QPR-J’s convergent validity.

The RAS is a user-rating measure examining personal recovery developed in the USA; a 41-item version and a 24-item version exist [9, 23]. Responses use a 5-point Likert scale (1 = *strongly disagree,* 5 = *strongly agree*). Higher scores indicate greater recovery. The validity and reliability of both versions of the RAS have been supported in the USA (Corrigan 1999, Corrigan 2004). The validity and reliability of the Japanese version of the 24-item RAS have been supported [24].

The Short Form-8 (SF-8) is a self-report measure that assesses health-related QOL; it contains subscales examining general physical health (the Physical Component Summary: PCS) and mental health (the Mental Component Summary: MCS). Higher scores indicate better QOL [25]. The validity and reliability of the SF-8–Japanese Version have been supported [26].

The QPR-J’s convergent validity was examined by calculating the Pearson product-moment correlation between the Japanese RAS and the Japanese SF-8’s mental health subscale (MCS). Values of 0.40—0.70 and >0.70 were considered moderate and strong, respectively.

#### Reliability testing

The test-retest reliability was assessed in a subsample of participants who completed the survey again after a two-week interval (*N* = 10). Intraclass correlation coefficients (ICC) were calculated as ICC (2, 1) using a two-way random effects model. We considered ICCs >0.80 to indicate excellent agreement [27].

Internal consistency reliability was examined using Cronbach’s alpha [28] calculated between scores on the full version, intrapersonal and interpersonal subscales. We considered alpha coefficients ≥0.70 satisfactory [29] and those ≥0.60 acceptable [30, 31].

#### Confirmatory factor analysis

We performed a confirmatory factor analysis (CFA) to test the fitness of the data to the factor structure extracted from the original 2-factor subscales of the English scale. The two factors were “the intrapersonal subscale” (1, 2, 3, 4, 5, 6, 7, 8, 9, 10, 11, 12, 13, 18, 19, 21, 22) and “the interpersonal subscale” (14, 15, 16, 17, 20). We reported the goodness of fit index including the comparative fit index (CFI) and the root mean square error of approximation (RMSEA). The acceptability of model fit was judged by the recommended standards: CFI > 0.90 and the RMSEA values of 0.06 or less for a good fit and 0.08 or less for a reasonable fit [32].

#### Exploratory factor analysis

We conducted an EFA along with the CFA to compare the original factor solution to the one that would emerge from the data. We performed an EFA to explore factor structure of the QPR-J and reported all statistical metrics (Cronbach’s alpha for internal consistency and the test-retest reliability) related to the yielded factors.

The QPR-J’s factorial validity was evaluated using maximum likelihood estimation with Promax rotation. Maximum likelihood estimation gives us the best results when the data are relatively normally distributed. ‘Maximum likelihood estimation allows for the computation of a wide range of indexes of the goodness of fit of the model [and] permits statistical significance testing of factor loadings and correlations among factors and the computation of confidence intervals’ [33].

Eigenvalues ≥1.00 were considered to indicate factors. Assumptions regarding matrix identity and sampling adequacy were evaluated using Bartlett’s Test of Sphericity and the Kaiser-Meyer-Olkin (KMO) test. Inter-factor correlations for the final model were examined to determine the extent of correlations among factors.

All analyses were conducted using IBM SPSS Statistics, version 22.0 (IBM SPSS, Armonk, NY, USA). The CFA was conducted using IBM SPSS AMOS, version 22.0 (IBM SPSS, Armonk, NY, USA).

## Results

We developed the QPR-J (cf. Additional file 1) and tested the validity of the QPR-J in a community sample of Japanese individuals. We excluded 21 respondents who did not respond to the QPR-J and analysed data from 197 respondents (90.4% of the initial 218 service users; Table 1). Respondents were mostly females (61.9%), never married (72.6%), living with their families, and diagnosed with schizophrenia (49.2%). Respondents’ average age was 42.0 ± 10.9 years. A subsample completed the QPR-J questionnaire again two weeks later to examine test-retest reliability (*N* = 10). They were mostly females (70.0%), were never married (50.0%), lived with their families, and had been diagnosed with schizophrenia (80.0%), and their average age was 44.7 ± 6.5 years.

**Table 1:** Demographic characteristics of the study participants

Characteristics		Test (*N* = 197)		Retest (*N* = 10)		Chi-squared test *(Mann-Whitney U test)* ^c^	
		N *(mean)*	% *(SD* ^a^)	N *(mean)*	% *(SD* ^a^)	chi-sq (*U*)	*p*-value
Age, years		*42.0*	*10.9*	*44.7*	*6.5*	*645.0*	0.31
	Missing	9		1		1	
Gender							
	Male	73	37.1	3	30.0	0.07	0.79
	Female	122	61.9	7	70.0		
	Missing	2	1.0	0	0		
Marital status							
	Unmarried	143	72.6	5	50.0	4.61	0.10
	Currently married	24	12.2	2	20.0		
	Divorced/widowed	26	13.2	3	30.0		
	Missing data	4	2.0	0	0		
Living situation^b^							
	Single	47	23.9	3	30.0		
	With parents	97	49.2	5	50.0		
	With sibling(s)	29	14.7	2	20.0		
	With partner	16	8.1	2	20.0		
	With child	11	5.6	1	10.0		
	Other	27	13.7	0	0		
	Missing	3	1.5	0	0		
Classification of mental disorder^b^							
	Mental disorders due to psychoactive substance use	21	10.7	0	0		
	Schizophrenia	97	49.2	8	80.0		
	Mood disorders	68	34.5	1	10.0		
	Anxiety, Adjustment disorders	19	9.6	0	10.0		
	Intellectual disabilities	4	2.0	1	10.0		
	Developmental disorders	16	8.1	1	10.0		
	Epilepsy	10	5.1	0	0		
	Other	21	10.7	1	10.0		
	Not known	9	4.6	0	0		
	Missing	5	2.5	0	0		
Duration of the current service							
	Months	*43.7*	*59.0*	*30.86*	*26.3*	*613.50*	0.91
	Missing	10		3		3	

^a^: Standard deviation

^b^: Total percentage will exceed 100 due to multiple responses

^c^: Chi-squared and Mann-Whitney U tests were used to compare the proportions of the demographic characteristics between participants and non-participants in the retest

Table 2 presents mean scores for the examined variables. Table 3 presents QPR-J item-level ratings. The QPR-J items 9 and 13 were rated slightly higher than the other items.

**Table 2:** Sample scores on the study measures (*N* = 197)

Scale^a^	N	Range	Mean	Standard Deviation
QPR-J	197	4–86	56.8	12.8
Japanese RAS	187	24–119	82.1	15.2
Japanese SF-8 (PCS)	190	4–21	9.6	3.6
Japanese SF-8 (MCS)	193	4–20	10.6	3.7

^a^: RAS: the Recovery Assessment Scale

SF-8 (PCS): Short Form-8 (Physical Component Summary)

SF-8 (MCS): Short Form-8 (Mental Component Summary)

**Table 3:** Item-level rating on the QPR-J (*N* = 197)

No.	Item	Mean	Standard Deviation	Median	IQR^a^
1	I feel better about myself	2.51	0.98	3	2–3
2	I feel able to take chances in life	2.41	1.16	2	2–3
3	I am able to develop positive relationships with other people	2.48	0.90	3	2–3
4	I feel part of society rather than isolated	2.24	1.07	2	2–3
5	I am able to assert myself	2.46	0.92	3	2–3
6	I feel that my life has a purpose	2.58	1.12	3	2–3
7	My experiences have changed me for the better	2.92	0.90	3	3–4
8	I have been able to come to terms with things that have happened to me in the past and move on with my life	2.68	0.98	3	2–3
9	I am basically strongly motivated to get better	3.37	0.79	4	3–4
10	I can recognise the positive things I have done	2.40	1.04	2	2–3
11	I am able to understand myself better	2.72	0.88	3	2–3
12	I can take charge of my life	2.28	1.03	2	2–3
13	I am able to access independent support	3.09	0.87	3	3–4
14	I can weigh up the pros and cons of psychiatric treatment	2.44	1.00	2	2–3
15	I feel my experiences have made me more sensitive towards others	2.61	0.92	3	2–3
16	Meeting people who have had similar experiences makes me feel better	2.87	0.99	3	2–4
17	My recovery has helped challenge other peoples views about getting better	2.24	0.95	2	2–3
18	I am able to make sense of my distressing experiences	2.34	1.11	2	2–3
19	I can actively engage with life	2.45	1.08	3	2–3
20	I realise that the views of some mental health professionals is not the only way of looking at things	2.72	0.96	3	2–3
21	I can take control of aspects of my life	2.28	0.93	2	2–3
22	I can find the time to do the things I enjoy	2.77	0.96	3	2–3

^a^: IQR: Interquartile range

### Validity testing

The scores on the total QPR-J, intrapersonal and interpersonal subscales were significantly and positively correlated with the scores on the RAS and SF-8 MCS (Table 4). These results indicated adequate convergent validity for each scale.

**Table 4:** Pearson’s correlation coefficients on the QPR-J intrapersonal (17 items), QPR interpersonal (5 items)

Scale^a^	Full-version	Intrapersonal subscale	Interpersonal subscale
Japanese RAS	0.77**	0.78**	0.54**
Japanese SF-8 (PCS)	0.10	0.11	0.05
Japanese SF-8 (MCS)	0.25**	0.28**	0.16**

^a^: RAS: the Recovery Assessment Scale

SF-8 (PCS): Short Form-8 (Physical Component Summary)

SF-8 (MCS): Short Form-8 (Mental Component Summary)

**p < 0.01

### Reliability testing

The ICC values for the full version (0.85 [95% CI 0.35–0.97]), intrapersonal subscale (0.85 [95% CI 0.35–0.97]), and interpersonal subscale (0.89 [95% CI 0.55–0.97]) were satisfactory.

Cronbach’s alpha coefficients indicated excellent internal consistency for the full version, intrapersonal subscale (α = 0.91 and 0.90 respectively). The internal consistency of the interpersonal subscale was acceptable but relatively low (α = 0.65).

### Confirmatory factor analysis

Goodness of fit index for the original 2-factor model were reasonable fit (CFI = 0.87 and RMSEA = 0.07) (Tables 5 and 6).

**Table 5:** Results of the confirmatory factor analysis: Goodness-of-fit indices for the two-factor QPR-J models

CFI	RMSEA	AIC	χ2	df	*p*
0.87	0.07	553.22	419.22	208.00	< 0.001

CFI: Confirmatory fit index

RMSEA: Root mean square error of approximation

AIC: Akaike information criterion

**Table 6:** Item loadings for the confirmatory factor analysis

		Two-factor model	
No	Item	Intrapersonal	Interpersonal
1	I feel better about myself	0.56	
2	I feel able to take chances in life	0.54	
3	I am able to develop positive relationships with other people	0.61	
4	I feel part of society rather than isolated	0.61	
5	I am able to assert myself	0.51	
6	I feel that my life has a purpose	0.68	
7	My experiences have changed me for the better	0.70	
8	I have been able to come to terms with things that have happened to me in the past and move on with my life	0.75	
9	I am basically strongly motivated to get better	0.27	
10	I can recognise the positive things I have done	0.68	
11	I am able to understand myself better	0.57	
12	I can take charge of my life	0.59	
13	I am able to access independent support	0.42	
18	I am able to make sense of my distressing experiences	0.64	
19	I can actively engage with life	0.78	
21	I can take control of aspects of my life	0.60	
22	I can find the time to do the things I enjoy	0.58	
14	I can weigh up the pros and cons of psychiatric treatment		0.56
15	I feel my experiences have made me more sensitive towards others		0.65
16	Meeting people who have had similar experiences makes me feel better		0.52
17	My recovery has helped challenge other peoples views about getting better		0.73
20	I realise that the views of some mental health professionals is not the only way of looking at things		0.22

### Exploratory factor analysis

An EFA was conducted using maximum likelihood estimation with Promax rotation. Bartlett’s Test of Sphericity (χ2 = 1, 756, *p* < 0.001) and the KMO test (0.90) indicated that the correlation matrix was factorable. Five factors had eigenvalues >1.0. Item factor loading was 0.34–0.70 which was not considered as good loadings. In addition, one factor (Factor 5) had only 2 item (Table 7). The five factors accounted for 47.64% of the total scale variance. Factor 1 (Positive relationships and redefining the meaning of life, 8 items) accounted for 17.79%, Factor 2 (Improving the skills of self-assessment and literacy of the treatment, 2 items) accounted for 20.61%, Factor 3 (Accepting the illness and positive decision making, 6 items) accounted for 3.53%, Factor 4 (Support from others and motivation to change, 4 items) accounted for 3.41% and Factor 5 (Self-management, 2 items) accounted for 2.30%.

**Table 7:** Item loadings for the exploratory factor analysis and statistical metrics (Cronbach’s alpha for internal consistency and ICC values for test-retest reliability)

			loading				
Intra/Inter	No	Item	Factor 1	Factor 2	Factor 3	Factor 4	Factor 5
			Positive relationships and redefining the meaning of life	Improving the skills of self-assessment and literacy of the treatment	Accepting the illness and positive decision making	Support from others and motivation to change	Self-management
Intra	4	I feel part of society rather than isolated	**0.76**	−0.11	−0.05	−0.04	0.13
Intra	5	I am able to assert myself	**0.69**	0.00	0.01	−0.17	−0.04
Intra	3	I am able to develop positive relationships with other people	**0.57**	−0.28	0.15	0.27	−0.01
Intra	6	I feel that my life has a purpose	**0.54**	0.18	0.21	−0.09	−0.15
Intra	10	I can recognise the positive things I have done	**0.44**	0.41	0.02	−0.13	−0.03
Inter	17	My recovery has helped challenge other peoples views about getting better	**0.44**	0.16	−0.15	0.37	0.00
Intra	18	I am able to make sense of my distressing experiences	**0.44**	0.43	−0.11	−0.04	−0.02
Intra	19	I can actively engage with life	**0.36**	0.10	0.26	0.15	0.09
Intra	11	I am able to understand myself better	−0.14	**0.78**	0.09	0.03	−0.01
Inter	14	I can weigh up the pros and cons of psychiatric treatment	−0.03	**0.69**	−0.05	0.01	0.08
Intra	22	I can find the time to do the things I enjoy	0.17	−0.11	**0.57**	−0.19	0.21
Intra	1	I feel better about myself	0.13	−0.08	**0.56**	0.09	−0.02
Intra	2	I feel able to take chances in life	0.11	0.29	**0.50**	−0.17	−0.12
Intra	8	I have been able to come to terms with things that have happened to me in the past and move on with my life	0.16	0.12	**0.42**	0.22	0.02
Intra	7	My experiences have changed me for the better	0.18	0.07	**0.39**	0.27	−0.02
Inter	20	I realise that the views of some mental health professionals is not the only way of looking at things	−0.21	0.02	**0.32**	0.11	0.13
Intra	9	I am basically strongly motivated to get better	−0.29	−0.05	0.13	**0.73**	−0.14
Inter	16	Meeting people who have had similar experiences makes me feel better	0.16	0.07	−0.18	**0.54**	−0.06
Intra	13	I am able to access independent support	0.15	−0.04	0.07	**0.37**	0.00
Inter	15	I feel my experiences have made me more sensitive towards others	−0.20	0.36	0.06	**0.36**	0.20
Intra	21	I can take control of aspects of my life	0.04	0.05	0.14	−0.17	**0.94**
Intra	12	I can take charge of my life	0.37	0.09	−0.12	0.17	**0.26**
	Cronbach’s alpha	0.89	0.72	0.82	0.87	0.80	
	ICC values	0.89	0.72	0.82	0.87	0.80	
	(95% CI)	(0.54–0.97)	(−0.11–0.93)	(0.21–0.96)	(0.48–0.97)	(0.18–0.95)	
	Extraction Sums of Squared Loadings						
	Total	3.91	4.53	0.78	0.75	0.51	
	% of variance explained	17.79	20.61	3.53	3.41	2.30	
	Cumulative % of variance explained	17.79	38.40	41.93	45.34	47.64	
	Rotation						
	Total	6.63	5.42	5.38	4.20	3.45	

Boldface type indicates the highest factor loading

## Discussion

This study aimed to develop and examine the validity and reliability of the QPR-J. The results indicated adequate convergent validity. The QPR-J’s full version, and intrapersonal subscale showed good test-retest reliability and excellent internal consistency reliability.

The scores on the total QPR-J, intrapersonal, and interpersonal subscales were significantly and positively correlated with the scores on the RAS-J (*r* = 0.77, 0.78, and 0.54, respectively), which is slightly above the correlations reported in a previous study reported between the QPR and RAS (*r* = 0.73, 0.75, and 0.46, respectively) [13]. The scores on the total QPR-J were also significantly and positively correlated with the scores on the SF-8 MCS, which is in line with previous studies reporting correlations between the original QPR and the Personal and Social Performance Scale (including socially useful activities and personal and social relationships) [12], the QPR-Chinese and Schizophrenia Quality of Life scale [16], and the QPR-Swedish and General Quality of Life [34]. These results indicate that the QPR-J has satisfactory convergent validity.

The results also indicated that the QPR-J has satisfactory test–retest reliability (ICC values for the full version: 0.85 [95% CI 0.35–0.97], intrapersonal subscale: 0.85 [95% CI 0.35–0.97], and interpersonal subscale: 0.89 [95% CI 0.55–0.97]). Test–retest reliability of the original QPR (*n* = 88) was ‘fair to good’ for the total QPR (ICC = 0.74, 95% CI 0.63–0.82) and ‘good’ for intrapersonal (ICC = 0.75, 95% CI 0.64–0.83) and interpersonal (ICC = 0.66, 95% CI 0.53–0.77). The test–retest assessments in our study were performed with a small sample size (*N* = 10). Therefore, future validation studies of the Japanese version of the questionnaire should better assess the test–retest reliability of the measure. Future validation studies of the QPR-J with large sample sizes sufficient to evaluate test–retest reliability are needed.

Cronbach’s alpha coefficients indicated excellent internal consistency for the full version and intrapersonal subscale (α = 0.91 and 0.90, respectively). The internal consistency of the interpersonal subscale was acceptable but relatively low (α = 0.65). These results were consistent with internal consistency of the original QPR (α = 0.89, 0.90, and 0.49). Previous studies indicated excellent internal consistency for the full version (QPR-Chinese: α = 0.90 and QPR-Swedish: α = 0.91). An independent evaluation of the psychometric properties of the original QPR [13] recommended 15-item versions excluding the QPR interpersonal subscale for use in research and clinical practice because of the poor internal consistency of the QPR interpersonal subscale. Further research to evaluate the psychometric properties of QPR-J with a sufficient sample size is needed.

An EFA of the original 22-item version identified two factors; these were labelled ‘intrapersonal’ and ‘interpersonal’ [10]. The EFA supported a 3-factor structure (Self-Empowerment, Effective Interpersonal Relationships, and Rebuilding Life) [16] in the QPR-Chinese and one-factor structure summarized as intrapersonal factors in the QPR-Swedish [34]. An EFA of the QPR-J identified five factors. When compared with the two factors of the original scale [10], the ‘Interpersonal’ subscale of the original QPR was divided into two factors; ‘Factor 1: Positive relationships and redefining the meaning of life’ (Item 17) and ‘Factor 4: Support from others and motivation to change’ (Item 15 and 16). Further, the attitude of Japanese people toward others and toward their life may be divided into ‘active relationships’ (Positive relationships and redefining the meaning of life) and ‘passive relationships’ (Support from others and motivation to change). Cultural differences in values of social relationships might affect these classifications. Japanese are strongly motivated toward relational harmony and interdependence [35]. An EFA of the QPR-J extracted ‘recovery process through positive relationship with others (Factor 1)’ and ‘recovery process within support from others (Factor 4)’. Japanese may build positive relationships (items 4, 5, 3, and 17) and recognise and define the meaning of life through the relationship (items 6, 10, 18, and 19) [Factor 1]. In addition, belongingness and support from others (item 16 and 13) and support for others (item 15) might motivate them to change (item 9) [Factor 4]. Some of the factor loadings were less than .50, which indicated poor factor loading, and the five factors accounted for 47.64% of the total scale variance. These trends of poor loadings did not change even after limiting the diagnosis to schizophrenia and/or mood disorders (Additional file 2: Table S1 and Table S2). Further research with a large sample size sufficient to allow EFA sufficiently is needed.

The result of the CFA indicated reasonable goodness of fit indexes for the original 2-factor model. Therefore, we adopted the factor structure extracted from the original 2-factor based on our CFA results. Some of the items had a factor loading below 0.5 in the CFA. Item 20 (‘I realise that the views of some mental health professionals is not the only way of looking at things’) had a factor loading 0.22 in the QPR-J and 0.38 in the QPR [13]. It may be too vague and difficult for the participants to think about “some mental health professionals” and “way of looking at things”. Item 13 (‘I am able to access independent support’) also had low factor loadings in the QPR-J (0.27). All the participants were recruited from community mental health services, and most used independent support. A careful review of item wording and diversity of sampling is needed in a replication study.

The first research examining recovery was qualitative and originated in the Western Europe and North America. Some studies that examined recovery have discussed international differences in the conceptualisation of personal recovery [36, 37]. A cross-cultural study examining recovery using the RAS found that “personal confidence and hope” and “reliance on others” varied in conceptualisation between the USA and Japanese samples [38]. Another cross-cultural study examining well-being found that personal control and relational harmony most strongly predicted well-being in the USA and Japan, respectively [39]. A cultural comparison study suggested that patients from Western European and Japanese cultures have different typical recovery needs [40]. Future research should further examine the Japanese conceptualisation of recovery and well-being.

Here are some of the comments we received from the participants: “I think that answering the QPR-J was a good opportunity to think about myself”, ‘I hope the staff will receive recovery oriented-education and provide better services’. Based on such comments, we believe that researchers should assess the process of recovery using the QPR-J and implement the best practices of recovery-oriented services in Japan.

This study has the following limitations. First, we drew respondents from suburbs in specific urban areas and using mental health services; therefore, the sample may not accurately represent the Japanese population. Further research is needed with more diverse samples. Second, Japan and the UK may have importantly different cultural and mental health care contexts. Future research should develop or adapt questionnaires to suit the Japanese cultural context. Finally, as we mentioned above, future validation studies with large sample sizes sufficient to evaluate test–retest reliability and to allow sufficient EFA of the QPR-J are needed.

## Conclusions

The QPR-J is valid, and it reliably measures recovery among mental health service users in Japan.

## Additional files

Additional file 1: Japanese version of the Questionnaire about the Process of Recovery (QPR-J). (PDF 133 kb)

Additional file 2: Table S1. Item loadings for the exploratory factor analysis and statistical metrics with people with schizophrenia. **Table S2.** Item loadings for the exploratory factor analysis and statistical metrics with people with schizophrenia and/or mood disorder. (XLSX 20 kb)

## Abbreviations

ICC: Intraclass correlation coefficients
KMO: Kaiser-Meyer-Olkin
QOL: Quality of life
QPR: Questionnaire about the process of recovery
RAS: Recovery assessment scale
SF-8 (MCS): Short form-8 (Mental component summary)
SF-8 (PCS): Short form-8 (Physical component summary)
