# Correction to: The Japanese version of the questionnaire about the process of recovery: development and validity and reliability testing

**DOI:** 10.1186/s12888-020-2430-y

**Published:** 2020-01-09

**Authors:** Akiko Kanehara, Risa Kotake, Yuki Miyamoto, Yousuke Kumakura, Kentaro Morita, Tomoko Ishiura, Kimiko Shimizu, Yumiko Fujieda, Shuntaro Ando, Shinsuke Kondo, Kiyoto Kasai

**Affiliations:** 1 Department of Neuropsychiatry, Graduate School of Medicine, The University of Tokyo, 7-3-1 Hongo, Bunkyo-ku, Tokyo, 113-8655 Japan; 2 Seiwa-Kai Nishikawa Hospital, 293-2 Minatomachi, Hamada-shi, Shimane 697-0052 Japan; 3 Department of Mental Health /Psychiatric Nursing, Graduate School of Medicine, The University of Tokyo, 7-3-1 Hongo, Bunkyo-ku, Tokyo, 113-0033 Japan; 4 Department of Rehabilitation, The University of Tokyo Hospital, 7-3-1 Hongo, Bunkyo-ku, Tokyo, 113-8655 Japan

**Correction to: BMC Psychiatry**

**https://doi.org/10.1186/s12888-017-1520-y**

After publication of our article [1] we were notified that the 5-point Likert-type scale in Additional file 1 needs to change from “1 to 5” to “0 to 4”. The updated Additional file 1 is included in this correction.

In the original QPR (Neil et al., 2009), the scale ranged from 0 to 4. However, when the authors made the test version of the questionnaire form (in Japanese), it unintentionally became ranged from 1 to 5.

## Supplementary information

**Additional file 1.** Japanese version of the Questionnaire about the Process of Recovery (QPR-J).
