# Supplementary material for: The Japanese version of the questionnaire about the process of recovery: development and validity and reliability testing
Source: BMC Psychiatry. 2017 Nov 7;17:360. doi: 10.1186/s12888-017-1520-y (PMC5678745; doi:10.1186/s12888-017-1520-y)
Supplement: Supplementary file 1 — Japanese version of the Questionnaire about the Process of Recovery (QPR-J). (PDF 133 kb) [file 12888_2017_1520_MOESM1_ESM.pdf]

Additional file 1: Japanese version of the Questionnaire about the Process of Recovery (QPR-J)

日本語版リカバリープロセス尺度（QPR-J）

この調査では「リカバリー」についてお聞きします。「リカバリー」は、「精神的健康に困難を抱えながらも、夢や希望をもち、社会の中で自分の人生に新たな目的や意義を発展させ、主体的に生きることをさし、個人的で独自な過程」といわれています。リカバリーについて、現在、特にここ7日間のあなたの状態を思い浮かべてください。あなたの体験を最も表している番号に○をつけてください。

リカバリーについて、現在、特にここ7日間のあなたの状態を思い浮かべてください。あなたの体験を最も表している番号に○をつけてください。		全くそう思わない	そう思わない	どちらとも言えない	そう思う	とてもそう思う
1	自分自身のことを以前よりも良く思える	1	2	3	4	5
2	人生で思い切って何かをやってみようと思える	1	2	3	4	5
3	周りの人とプラスになる人間関係を築くことができる	1	2	3	4	5
4	社会とのつながりがないというよりも社会の一員だと感じている	1	2	3	4	5
5	自分の意見をちゃんと伝えることができる	1	2	3	4	5
6	自分の人生には意味があると感じている	1	2	3	4	5
7	これまでの経験で成長することができた	1	2	3	4	5
8	これまで自分に起きたことを受け入れて、前に進めるようになった	1	2	3	4	5
9	もっと元気になりたいと強く思っている	1	2	3	4	5
10	自分がしたよいことを思い返すことができる	1	2	3	4	5
11	自分自身のことを以前よりも理解することができるようになった	1	2	3	4	5
12	自分の生活に責任を持つことができる	1	2	3	4	5
13	支援機関（就労支援施設・相談支援機関など）を利用することができる	1	2	3	4	5
14	精神科での治療のメリット・デメリットを比べて選ぶことができる	1	2	3	4	5
15	自分の経験を通して、以前よりも思いやりのある人間になったと感じる	1	2	3	4	5
16	似たような経験をした人たちと会うと気持ちが楽になる	1	2	3	4	5
17	私の「リカバリー」体験は元気になることに対する周りの人のイメージを変える一助となった	1	2	3	4	5
18	自分のつらかった経験の意味を見出すことができる	1	2	3	4	5
19	前向きに人生に取り組むことができる	1	2	3	4	5
20	専門職（医師・看護師・心理士・精神保健福祉士など）の見方が、物事の考え方のすべてではないと思う	1	2	3	4	5
21	自分の様々な生活場面を自分でコントロールできる	1	2	3	4	5
22	楽しいことをする時間をつくることができる	1	2	3	4	5
